# Before attributing facial diplegia to neuroborreliosis, a plethora of differential diagnoses must be ruled out

**DOI:** 10.3325/cmj.2024.65.68

**Published:** 2024-02

**Authors:** Josef Finsterer

**Affiliations:** Neurology Department, Neurology & Neurophysiology Center, Vienna, Austria

*fifigs1@yahoo.de*

To the Editor,

We read with interest the case report by Blažina et al on a 68-year-old woman who developed isolated bilateral peripheral facial palsy with proven post-acute SARS-CoV-2 infection syndrome and neuroborreliosis (1). Since bilateral facial palsy disappeared within two weeks after starting ceftriaxone treatment (1), it was attributed to neuroborreliosis (1). The report is impressive, but several points require discussion.

The major limitation of the case report is that alternative causes of bilateral facial palsy were poorly ruled out. In addition to viral infections (cytomegalovirus, Epstein-Barr virus, varicella zoster virus, herpes simplex 1 and 2, HIV, and SARS-CoV-2), bacterial infections (*Leptospira*, *Borrelia burgorferi*, *Klebsiella pneumoniae*, *Toxoplasma gondii*), Guillain-Barré syndrome, leukemia, sarcoidosis, cranial base fracture, intracranial neoplasms, idiopathic cranial neuropathy, Melkerson-Rosenthal syndrome, demyelination diseases, Kawasaki disease, and post-vaccination syndrome (1), bilateral facial palsy can be attributed to a number of other diseases (Table 1) (2).

**Table 1:** Some possible causes of bilateral facial palsy

Autoimmune diseases	chronic inflammatory demyelinating polyneuropathy, Wegener's granulomatosis, Sjögren's syndrome, myeloperoxidase-related vasculitis, myasthenia gravis, acute disseminated encephalomyelitis, herb assumption, systemic lupus erythematosus
Infectious diseases	syphilis, masked mastoiditis, tick-borne meningitis, *Mycobacterium leprae*, botulism, cerebral toxoplasmosis, cryptococcal meningitis, enterovirus, d68 infection, HEV infection, granulocytic ehrlichiosis, Japanese encephalitis, leptospirosis, otitis media due to methicillin-resistant *Staphylococcus aureus*, *Mycoplasma pneumoniae*, malaria, scrub typhus, tuberculous meningitis, acute otitis media
Trauma	traumatic brain injury, bilateral condylar or mandibular fractures
Neoplasias	leukemia, lymphoma, pontine glioma, epidermoid cancer, ependymoma, metastasis, bilateral parotid gland carcinoma, unknown primary carcinoma
Iatrogenic causes	transparotid approach for bilateral condylar fracture, coagulopathy caused by antileukemic treatment, acute methotrexate encephalopathy after intraventricular methotrexate for lymphatic leukemia), metabolic disease (diabetes, renal osteodystrophy)
Vascular diseases	basilar artery thrombosis, pontine hemorrhage
Miscellaneous conditions	Foix-Chavany-Marie syndrome, multiple cranial neuropathy, brainstem encephalitis, benign intracranial hypertension, bulbospinal neuronopathy, Cogan syndrome, osteopetrosis, cholesteatoma, Bell’s palsy, familial Bell’s palsy

A second point is that bilateral facial palsy is not an extremely rare disease, as stated in the article (1). Bilateral facial palsy occurs in 0.3 to 2% of patients with facial palsy (3). In half of patients with bilateral facial palsy, other cranial nerves are involved (3). It should be clearly stated whether the index patient was thoroughly examined for involvement of cranial nerves other than the facial nerves.

A third point is that no information was provided as to whether the patient received the SARS-CoV-2 vaccination and how long the latency period between vaccination and the onset of bilateral facial palsy was. SARS-CoV-2 vaccination has been reported to cause facial diplegia (4).

A fourth point is that IgG and IgM antibodies and Western blot for borreliosis were not repeated after ceftriaxone to assess whether IgM levels returned to normal. The persistence of elevated IgM levels would indicate false-positive results.

A fifth point is that false-positive results of the *Borrelia* test were not taken into account. Ceftriaxone may be effective not only for neuroborreliosis but also for other bacterial or protozoal infections with *Staphylococcus aureus, Streptococcus pyogenes*, *Streptococcus pneumoniae*, *Moraxella catarrhalis*, *Enterobacteriaceae*, *Haemophilus influenzae*, *Escherichia coli*, and several others.

In summary, the excellent study has limitations, which complicate the interpretation of the results. Addressing these limitations could strengthen and reinforce the conclusion of the study.

## References

[1] Blažina K Martinez I Foro Znika M Bilateral facial nerve palsy as a presentation of coexisting neuroborreliosis and post-acute COVID-19 syndrome. Croat Med J 2023 64 440 3 10.3325/cmj.2023.64.440 38168526 PMC10797239

[2] Jung J Park DC Jung SY Park MJ Kim SH Yeo SG Bilateral facial palsy. Acta Otolaryngol 2019 139 934 8 10.1080/00016489.2019.1651134 31430217

[3] Molinari G Lucidi D Fernandez IJ Barbazza A Vanelli E Lami F Acquired bilateral facial palsy: a systematic review on aetiologies and management. J Neurol 2023 270 5303 12 10.1007/s00415-023-11897-7 37523065 PMC10576676

[4] Rossetti A Gheihman G O’Hare M Kosowsky JM Guillain-Barré syndrome presenting as facial diplegia after COVID-19 vaccination: a case report. J Emerg Med 2021 61 e141 5 10.1016/j.jemermed.2021.07.062 34538679 PMC8346349

